# Carrier Diversity Incorporation to Low-Complexity Near-ML Detection for Multicarrier Systems over V2V Radio Channel

**DOI:** 10.3390/s21186067

**Published:** 2021-09-10

**Authors:** Jose Alberto Del Puerto-Flores, Fernando Peña-Campos, Ramón Parra-Michel, Carolina Del-Valle-Soto

**Affiliations:** 1 Facultad de Ingeniería, Universidad Panamericana, Álvaro del Portillo 49, Zapopan, Jalisco 45010, Mexico; jpuerto@up.edu.mx; 2 Department of Electrical Engineering, Communications Section, CINVESTAV-IPN, Guadalajara, Jalisco 45019, Mexico; fernando.pena@cinvestav.mx (F.P.-C.); ramon.parra@cinvestav.mx (R.P.-M.)

**Keywords:** DFTS-OFDM, ICI mitigation, Near-ML, OFDM, OSIC, V2V

## Abstract

Inter-carrier interference (ICI) in vehicle to vehicle (V2V) orthogonal frequency division multiplexing (OFDM) systems is a common problem that makes the process of detecting data a demanding task. Mitigation of the ICI in V2V systems has been addressed with linear and non-linear iterative receivers in the past; however, the former requires a high number of iterations to achieve good performance, while the latter does not exploit the channel’s frequency diversity. In this paper, a transmission and reception scheme for low complexity data detection in doubly selective highly time varying channels is proposed. The technique couples the discrete Fourier transform spreading with non-linear detection in order to collect the available channel frequency diversity and successfully achieving performance close to the optimal maximum likelihood (ML) detector. When compared with the iterative LMMSE detection, the proposed system achieves a higher performance in terms of bit error rate (BER), reducing the computational cost by a third-part when using 48 subcarriers, while in an OFDM system with 512 subcarriers, the computational cost is reduced by two orders of magnitude.

## 1. Introduction

The development of vehicle-to-vehicle (V2V) wireless communications has experienced a boom in recent years due to its main applications for traffic control and road safety, such as: reducing traffic in main avenues, collision prevention, autonomous vehicle development, remote tracking of vehicles, etc. Different measurement campaigns and channel sounding in the V2V environment [1,2] confirm the existence of high Doppler spread frequencies (above 600 Hz), which causes inter-carrier interference (ICI) to be one of the main problems that affect the receivers’ performance and greatly complicate the estimation and data detection tasks.

The specific problem of channel parameter estimation at the receiver is further complicated in V2V systems because the ICI also affects the pilot sub-carrier integrity required to properly carry out the channel estimation. Among the works that tackle this problem, the most relevant are [3,4]; these works propose an iterative receiver with a channel estimator based on a two-dimensional basis expansion model (2D-BEM). However, one problem with these approaches is that they only reconstruct the channel variations at an OFDM frame resolution, omitting the temporal variation of the channel within one OFDM symbol. In addition, these works employ a linear data detection scheme that is unable to mitigate ICI in highly time-variant channels. Furthermore, the iterative receivers in [3,4], require at least 5 iterations to deliver acceptable performance in terms of bit error rate (BER). In [5], channel estimation and tracking are performed by resolving dominant multipath components.

Approaches [6,7,8] make a substantial reduction in the computational complexity required in the data detection. This is achieved by approximating the original signal model using a reduced signal model in the frequency domain, based on the band equalization, where the channel matrix is truncated to keeping only a small number of bands. The approximate observation model described in [6,7,8] does not include the channel frequency diversity, which causes the linear detection the achieving of a lower performance compared to the detection carried out in the complete observation model. Furthermore, the approximate model described in [6,7,8] is not compatible with non-linear detectors when including the channel frequency diversity.

Works [9,10] presents systems with data estimators suitable to counteract the distortions produced by the ICI, achieving better performance than conventional receivers. However, they modify the physical layer of the 802.11p standard in order to introduce additional training sequences, which decreases the spectral efficiency of the system and represents an incompatibility problem with the 802.11p standard [11]. They also use channel estimators with an observation window that covers a large number of OFDM symbols, increasing the memory required for their implementation and the system’s latency.

Since data detection is the process with the greatest computational complexity in the receiver, the practical feasibility of any system designed to work in real-time in V2V channels depends mainly on the order of complexity required in the detection task. The computational complexity of the optimal detector of maximum likelihood (ML) is $O(N_{D}\Omega^{N_{D}})$[12], being $N_{D}$ number of data subcarriers and Ω the constellation size. This complexity makes the real-time implementation to be less viable in comparison with linear detectors whose complexity is bounded in $N_{D}^{3}$.

Recently, a series of detectors have been proposed, which have lower complexity than the ML detector while maintaining similar performance. The spherical detector (SD) [13], which is based on tree-search algorithms, has been applied to multicarrier systems [14], yielding lower complexity than the ML detection [15], as it was expected. Some non-linear detection schemes are based on the M-algorithm, and the QR decomposition of the channel matrix [16,17]. Ordered successive iterative cancellation (OSIC) has been proposed recently in [18]. However, all the aforementioned non-linear detectors are applied to a system model that does not include the channel frequency diversity, as well as not exploiting an approximation of the band channel matrix during its QR decomposition. However, all the aforementioned non-linear detectors are applied to a system model that does not include the channel frequency diversity, as well as not exploiting an approximation of the band channel matrix during its QR decomposition.

An important factor that impacts the multicarrier system performance is the frequency selectivity of the channel. This characteristic of the broadband channel makes it difficult to recover some signals degraded by a deep fading of the channel. Because the non-linear data detection is carried out consecutively, the channel’s selectivity causes degradation in performance of the non-linear detection, even in conditions of high signal-to-noise ratio (SNR). The works mentioned above [3,4] tackle this problem through the inclusion of a stage for channel coding, with the consequent loss of spectral efficiency. In recent works [19,20,21], it is observed that low-density parity-check (LDPC) codes have a better performance on doubly selective channels compared to convolutional codes and turbo coding. In LDPC coded transmission schemes, the system parameters are usually optimized towards a particular scenario to achieve capacity-approaching performance. However, due to high mobility in V2V communications, several channel scenarios can be experienced within the narrow time window. In order to maintain the performance of the LDPC encoded transmission schemes, the system parameters must be individually optimized for different scenarios, which translates into an increase in the computational complexity of the system making real-time implementation difficult. Additionally, at the signal level, no schemes have been incorporated to effectively take advantage of the frequency selectivity in the form of diversity to achieve better performance in the non-linear detection without significantly affecting the system’s spectral efficiency.

### 1.1. Objectives and Contributions

This paper proposes a new detection algorithm that yields similar performance to that of ML with low computational complexity. A data precoding scheme is proposed that efficiently exploits the channel’s frequency diversity without affecting the system’s spectral efficiency. The proposed detector offers two main advantages:

- It assimilates the linear data precoding stage so that the operation is obtained with equivalent channel matrices that maintain the band structure. This makes it possible to extract the channel diversity, which significantly improves performance while demanding low complexity;
- It uses an improved search order that enables the decoder to find the optimal solution in fewer iterations. Furthermore, the detector’s maximum search size can be set to an amount of operations that will not have a significant impact on performance.

Finally, an iterative reception structure is proposed that makes it possible to eliminate ICI among data subcarriers. The performance achieved with this technique after one iteration comes close to that obtained in conditions of exact knowledge of the channel.

### 1.2. Abbreviations and Acronyms

Lower (upper) case letters refer to vectors (matrices); ${\left[\cdot \right]}^{T}$, ${\left(\cdot \right)}^{H}$, ${\left(\cdot \right)}_{N}$ and ${\left[\cdot \right]}_{T}$ are the transpose, Hermitian, circular shift modulo *N* and band truncation operators, respectively; ${\left(\cdot \right)}^{k}$ refers to the *k*-th OFDM symbol being considered. The subscripts ${\left(\cdot \right)}_{P}$ and ${\left(\cdot \right)}_{D}$ are the sampled versions of the vectors in the pilot and data positions; and in the case of the matrices, the versions sampled in the rows and columns in the pilot and data positions.

### 1.3. Organization

This paper is organized as follows: The signal model used is described in Section 2. The proposed reception system with the incorporation of frequency dispersion is addressed in Section 3. The low complexity non-linear detection proposed is described in Section 4. The complete block structure of the transmitter and receiver is presented in Section 5. Computational complexity analysis is addressed in Section 6. The simulation results are presented in Section 7. Finally, the conclusions are stated in Section 8.

## 2. System Model

**Figure 1:**
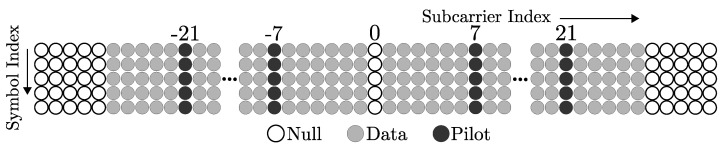
Pilot pattern in an 802.11p OFDM frame.

The frame structure in 802.11p consists of a preamble that contains 10 symbols, each one lasting 1.6 μs, found at the beginning of the frame and used to synchronize the system. Subsequently, two long training symbols are transmitted, each one lasting 6.4 μs, used for fine synchronization and channel estimation. The remaining part of the frame, which has a variable length, is used to transmit the payload. Depending on the different modulation and coding schemes used, IEEE 802.11p permits a number of data transmission speeds ranging from 3 to 27 Mbps [22]. The physical layer of 802.11p, shown in Figure 1, uses 64 subcarriers per OFDM symbol, including 48 data subcarriers, 4 pilot subcarriers located at the indexes [−21, −7],[7, 21], 11 virtual subcarriers, and one subcarrier for direct current (DC) component.

Let $x^{k}\left[n\right]$ the *k*-th transmitted OFDM symbol of $N_{b}=N+N_{g}$ samples, where *N* and $N_{g}$ are the number of subcarriers and the length of the cyclical prefix (CP), respectively. Assuming that the CP is long enough to absorb the channel impulse response (CIR), the *k*-th received symbol $y^{k}\left[n\right]$ after removing CP can be expressed in its complex baseband representation as:

(1) $$y^{k}\left[n\right]=\sum_{l=0}^{L-1}h^{k}\left[n,l\right]x^{k}[{\left(n-l\right)}_{N}]+w^{k}\left[n\right],$$

where n={0,…,N−1}, l={0,…,L−1}, *L* denotes the CIR length, h[n,l] is the CIR for the *k*-th block in the *n*-th time instant for an impulse function introduced *l* samples earlier, and w[n] is the circular and symmetrical additive white Gaussian noise (AWGN), with zero mean and variance $\sigma_{w}^{2}=N_{0}/2$.

The circular convolution between the CIR and $x^{k}\left[n\right]$ can be rewritten in matrix form as:

(2) $$y^{k}=H^{k}x^{k}+w^{k}$$

where

$$\begin{array}{cc} y^{k} & ={\left[\begin{array}{ccc} y^{k}\left[0\right] & \begin{array}{cc} y^{k}\left[1\right] & \end{array}\cdots & y^{k}\left[N-1\right] \end{array}\right]}^{T},\\ x^{k} & ={\left[\begin{array}{ccc} x^{k}\left[0\right] & \begin{array}{cc} x^{k}\left[1\right] & \end{array}\cdots & x^{k}\left[N-1\right] \end{array}\right]}^{T},\\ w^{k} & ={\left[\begin{array}{ccc} w^{k}\left[0\right] & \begin{array}{cc} w^{k}\left[1\right] & \end{array}\cdots & w^{k}\left[N-1\right] \end{array}\right]}^{T}; \end{array}$$

in addition, $H^{k}$ is an N×N matrix, indexed 0,1,…,N−1 on each dimension, whose elements are formed with the CIR coefficients as follows:

(3) $${\left[H^{k}\right]}_{n,n^{\prime }}=h^{k}\left[n,{\left(n-n^{\prime }\right)}_{N}\right],$$

where $n,n^{\prime }=\left\{0,1,\ldots ,N-1\right\}$ and CIR is assumed to be zero for ${\left(n-n^{\prime }\right)}_{N}>L-1$. The OFDM symbol received in frequency domain (FD) is obtained by multiplying both sides of (2) by the matrix of the normalized discrete Fourier transform (DFT):

(4) $${\left[F\right]}_{n,n^{\prime }}=\frac{1}{\sqrt{N}}e^{\left(-j2\pi nn^{\prime }/N\right)},$$

which gives the result:

(5) $$\begin{array}{cc} u^{k} & ={\mathrm{FH}}^{k}x^{k}+z^{k}, \end{array}$$

where $u^{k}$ is the DFT of the $y^{k}$ and $z^{k}$ is the DFT of noise sequence. Since matrix F is unitary, Equation (5) can be expressed as:

(6) $$\begin{array}{cc} u^{k} & =G^{k}s^{k}+z^{k}, \end{array}$$

where $s^{k}={\mathrm{Fx}}^{k}$ is the OFDM symbol transmitted in the frequency domain, consisting of the χ data vector with $N_{D}$ data symbols, the $s_{P}$ pilot vector with $N_{P}$ pilots symbols, and $N_{G}$ guard symbols. $G={\mathrm{FHF}}^{H}$ is the channel frequency matrix (CFM). When the Doppler propagation is insignificant, G is a diagonal matrix, and the system is ICI-free. In V2V environments, due to high mobility of both the transmitter and the receiver, the combined effect of Doppler shift for each of the individual received paths results in significant Doppler dispersion, causing the CIR to become time varying within an OFDM symbol and resulting in matrix G to have energy on the components outside of the diagonal, giving rise to ICI. An example of the case of the scattered matrix of the channel is shown in Figure 2.

## 3. Proposed Receiver

### 3.1. Channel Estimation

The reduced number of pilots in a single OFDM symbol and the fact that they experience ICI complicate the task of estimating the time-varying CIR. To counteract this, the observation model of the channel estimator is extended to a sliding window that includes adjacent OFDM symbols as follows:

(7) $$\left[\begin{array}{c} u^{k-1}\\ u^{k}\\ u^{k+1} \end{array}\right]=\left[\begin{array}{c} G^{k-1}s^{k-1}\\ G^{k}s^{k}\\ G^{k+1}s^{k+1} \end{array}\right]+\left[\begin{array}{c} z^{k-1}\\ z^{k}\\ z^{k+1} \end{array}\right]$$

where the superscript of each variable represents the position relative to the current *k* symbol. In order to carry out the channel estimation with this observation model, the algorithm proposed in [23] is used, where BEM is applied so as to obtain a compact representation of the CIR in the interval of the three OFDM symbols as follows:

(8) $$h\left[n,l\right]=\sum_{r=0}^{M_{\tau }-1}\sum_{q=0}^{M_{D}-1}\rho_{q,r}\phi_{q}^{I}\left[n\right]\phi_{r}^{II}\left[l\right]+\varepsilon \left[n,l\right],$$

where $\rho_{q,r}$ are the coefficients of the expression, $m=\left\{0,\ldots ,3N_{b}-1\right\}$, $M_{\tau }$ and $M_{D}$ are the number of functions used to expand the delay time domain and the time domain, respectively, and $\left\{\phi_{q}^{I}\left[n\right],\forall q\in \left[0,M_{D}-1\right]\right\}$, $\left\{\phi_{r}^{II}\left[l\right],\forall r\in \left[0,M_{\tau }-1\right]\right\}$ are the functions that expand the time domain and the delay time, respectively. Given that in V2V scenarios the Doppler and delay dispersion presents statistics within a very diverse set, the discrete prolate spheroidal sequences (DPSS) are used as base functions since they optimally concentrate the energy in a finite time and bandwidth window. The modeling error for this representation in subspace is concentrated in the term ε[n,l].

To determine the number of functions needed in each of the CIR domains, we use the approach proposed in [24]:

(9) $$\begin{array}{cc} M_{\tau } & =\left\lceil F_{s}\tau_{max}\right\rceil \;+\;1, \end{array}$$

(10) $$\begin{array}{cc} M_{D} & =\left\lceil 2f_{D}3N_{b}/F_{s}\right\rceil \;+\;1. \end{array}$$

where ⌈·⌉ denotes the upward rounding operator, $F_{S}$ is the system’s bandwidth, $\tau_{max}$ is the maximum time delay spread and $f_{D}$ is the maximum frequency Doppler spread.

The information from the BEM of channel in the frequency and frequency Doppler domains are found compactly in the doubly-indexed matrix:

(11) $$\Phi_{q,r}^{k}={\mathrm{FB}}_{q,r}^{k}F^{H},$$

with

(12) $${\left[B_{r,q}^{k}\right]}_{n,n^{\prime }}={\phi_{q}}^{I}\left[n+\left(k+1\right)\left(N+N_{g}\right)\right]{\phi_{r}}^{II}\left[{\left(n-n^{\prime }\right)}_{N}\right]$$

and the BEM coefficients given by:

(13) $$\rho_{q,r}=\sum_{m=0}^{3N-1}\sum_{l=0}^{L-1}h\left[m,l\right]{\bar{\phi }}_{q}^{I}\left[m\right]{\bar{\phi }}_{r}^{II}\left[l\right].$$

Substituting the channel for its BEM in (6) yields the expression:

(14) $$u^{k}=\sum_{q=0}^{M_{D}-1}\sum_{r=0}^{M_{\tau }}\rho_{q,r}\Phi_{q,r}^{k}s^{k}+z^{k},$$

Annexing this representation of the channel to the Equation (7) and considering only the positions where the transmitted and received pilots are found, yields the following:

(15) $$u_{P}=\Lambda \rho +\epsilon_{P},$$

where:

(16) $$\begin{array}{cc} \Lambda^{T} & ={\left[\begin{array}{ccc} {\Lambda^{k-1}}^{T} & {\Lambda^{k}}^{T} & {\Lambda^{k+1}}^{T} \end{array}\right]}^{T}, \end{array}$$

(17) $$\begin{array}{cc} \Lambda^{k} & =\left[\begin{array}{ccc} \Phi_{1,P}^{k}s_{P}^{k} & \Phi_{2,P}^{k}s_{P}^{k} & \Phi_{I,P}^{k}s_{P}^{k} \end{array}\right], \end{array}$$

(18) $$\begin{array}{cc} u_{P} & ={\left[\begin{array}{ccc} u{{}_{P}^{k-1}}^{T} & u{{}_{P}^{k}}^{T} & u{{}_{P}^{k+1}}^{T} \end{array}\right]}^{T}, \end{array}$$

(19) $$\begin{array}{cc} \rho & ={\left[\begin{array}{cccc} \rho_{0} & \rho_{1} & \begin{array}{ccc} \cdots & \rho_{i} & \cdots \end{array} & \rho_{I} \end{array}\right]}^{T}; \end{array}$$

the subscript P refers to the sub-sample of the vectors and matrices in the rows and columns corresponding to the pilots’ position. For reasons of simplification in the notation, the indexed variable $i=q+M_{D}\left(r-1\right)$ was used, where $0\leq r\leq M_{\tau }$ and $0\leq q\leq M_{D}-1$. $\epsilon_{P}$ is the vector that concentrates the noise contributions, modeling error and intersymbol interference in order to simplify the expressions.

It is assumed that the receiver has matrix Λ and the received vector $u_{P}$, such that the calculation of the channel’s estimated coefficient vector can be obtained by the least-squares (LS) algorithm [23]:

(20) $$\begin{array}{c} \hat{\rho }={\left(\Lambda^{H}\Lambda \right)}^{-1}\Lambda u_{P}. \end{array}$$

Once these coefficients are obtained, any of the representations, such as the time-varying impulse response and the channel transfer function, can be calculated directly by computing the weighted sum of the base functions. In this way, the frequency-Doppler and frequency response matrix can be calculated by using the expression:

(21) $${\hat{G}}^{k}=\sum_{i=0}^{I-1}{\hat{\rho }}_{i}\Phi_{i}^{k}.$$

### 3.2. DFT Dispersion

The V2V channel selectivity makes OFDM systems susceptible to detection errors because the instant power of some subcarriers can be low due to the deep fading, which makes it difficult to detect the transmitted data. To counteract this problem, the precoding by dispersion in frequency (Direct Fourier Transform Spreading: DFTS) technique is used in this work because it uniformly distributes each symbol’s energy over the entire bandwidth and because its implementation, by means of the Fast Fourier Transform (FFT), is low in complexity. This operation can be represented formally on the transmitter side as follows:

(22) $$s_{D}=F_{D}\chi .$$

This is to say, the elements transmitted in frequency in the data positions are built by applying the Fourier matrix to the data symbols in vector χ. The elements of the Fourier matrix are determined by:

(23) $${\left[F_{D}^{}\right]}_{d^{\prime },d}=1/\sqrt{N_{D}}e^{j2\pi d^{\prime }d/N_{D}},$$

where $d,d^{\prime }=\left\{0,1,\ldots ,N_{D}\right\}$. Due to the fact that the data detection will only be carried out in the data subcarrier indexes, the original signal model in (4) is reduced to the following expression:

(24) $$u_{D}=G_{D}F_{D}\chi +G_{D,P}s_{P}+z_{D},$$

where $u_{D}^{}$ and $z_{D}$ are the received signal vector and the noise vector, respectively, each in the position of the data subcarriers. The $G_{D}$ matrix is obtained by taking the rows and columns of the data carrier positions. The term $G_{D,P}s_{P}$ represents the interference in the carriers, with data from the pilot carriers.

### 3.3. Non-Linear Detection on DFTS-OFDM System

**Figure 3:**
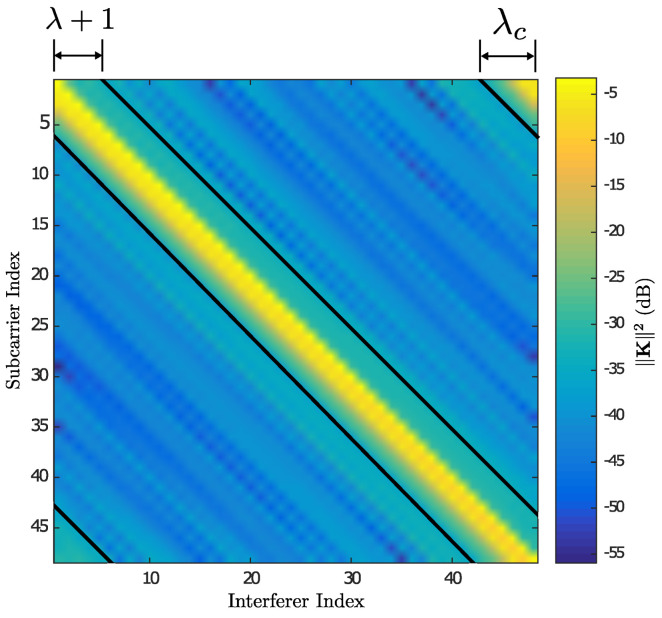
Plot of an example of ${∥K∥}^{2}$ in dB, where the quasi-band structure of K in V2V-DSC allows application of a matrix truncation with λ+1 strips.

The main contribution in this paper is the efficient integration of the DFTS precoding in the non-linear data detection process in the receiver. This has to be emphasized: one of the problems when using DFTS in the receiver is the difficulty of applying the non-linear detection algorithms while maintaining low computational complexity, since the equivalent channel matrix $G_{D}^{}F_{D}$ does not conserve a band structure. One solution to this issue is to find an operator that, upon being applied to the received signal, re-establishes the equivalent channel matrix’s band structure. In this sense, the inverse Fourier transform $F_{D}^{H}$ is used to complete the Cramer–Loève operator in the $G_{D}^{}$ channel matrix. In mathematical terms, the vector received in the data position is obtained as:

(25) $$\begin{array}{cc} F_{D}^{H}u_{D}^{} & =F_{D}^{H}G_{D}^{}F_{D}\chi +F_{D}^{H}G_{D,P}^{}s_{P}^{}+F_{D}^{H}z_{D}, \end{array}$$

(26) $$\begin{array}{cc} v & =K\chi +F_{D}^{H}G_{D,P}^{}s_{P}^{}+z_{D}, \end{array}$$

where $v=F_{D}^{H}u_{D}^{}$, $\chi =F_{D}^{H}s_{D}$ and $K=F_{D}^{H}G_{D}^{}F_{D}$ is the equivalent channel matrix after applying the inverse Fourier transform to the received symbols in the data position with linear precoding. In order to simplify the notation, the noise vector $z_{D}=F_{D}^{H}z_{D}$ maintains the same nomenclature since the orthonormal transforms does not affect its statistics. Notice that the correlation characteristics and quasi-band structure of matrix $G_{D}^{}$ imply that matrix K also possess a quasiband structure after the transformation, as shown in Figure 3. This structure in particular allows detection algorithms to diminish their computational complexity, while performing close to the ML detector in terms of BER.

#### Maximum Likelihood Detection Criteria

The data detection using the maximum likelihood criterion can be determined from the Equation (26) by finding the vector χ that minimizes the following metrics:

(27) $$\hat{\chi }=\underset{\chi \in \Omega^{N_{D}}}{\mathrm{argmin}}{∥v-K\chi ∥}^{2},$$

where Ω is the constellation used for data modulation and $\Omega^{N_{D}}$ is the set containing all possible combinations of the symbols transmitted through the $N_{D}$ data subcarriers. This data detection method is highly computationally complex due to the exhaustive calculation of all of the Euclidean distances that are needed to estimate the vector $\hat{\chi }$. In order to reduce the complexity required by the detection, it can be applied a truncation to the matrix K, keeping *B* non-zero diagonals. These are determined using the following equation:

(28) $$B=2(\lambda +\lambda_{c})+1,$$

where the *B* diagonals are distributed in 3 bands, one band is formed by the main diagonal and 2λ adjacent diagonals, the second band is formed by the $\lambda_{c}$ diagonals located in the upper right corner, and the third band is formed by $\lambda_{c}$ diagonals located in the lower-left corner. The rest of the diagonals are truncated to zero. The structure of the truncated K matrix is shown in Figure 3.

### 3.4. ICI Mitigation

As in related works [3,23,25], an iterative receiver is proposed, in which the estimated data can be reused to perform interference cancellation in the data, attaining an improved channel estimation in the next iteration. Mathematically, the estimated data in a it-th iteration can be described recursively as:

(29) $$u_{it}^{k}=u_{it-1}^{k}-{\left[{\hat{G}}_{it-1}^{k}\right]}_{T}s_{it-1}^{k},$$

where the sub-index it={0,1,2,…} denotes the iteration number; $u_{it}^{k}$ is the *k*-th received signal, ${\left[{\hat{G}}^{k}\right]}_{T}$ is the estimated channel matrix with null elements in the main diagonal and $s_{it-1}^{k}$ the received signal of the previous iteration.

## 4. Low Complexity Non-Linear Detection

The solution in (27) of the proposed signal model (26), although optimal, is not practical for implementation due to its high computational complexity. This section describes a methodology for non-linear detection of linearly precoded data; suitable for the signal model described in (26). The detection process consists of two stages; first, the sorted QR decomposition of the channel matrix K is performed. Subsequently, with the help of this decomposition, the proposed non-linear detectors of low computational complexity are executed. Each of the above algorithms is now described.

### 4.1. Sorted QR Decomposition

QR decomposition is used to obtain the matrices Q and R from the channel matrix with precoding K. This decomposition must comply with the following relationship:

(30) KP=QR,

where Q is an orthonormal matrix that fulfils the property $Q^{H}Q=I$, R is a superior triangular matrix, and P is a permutation matrix whose reordering depends on the signal to interference ratio. This decomposition can be accomplished using different methods; however, for this article, the method based on the Givens unit rotations is used [26]. In each step of the orthonormalization process for determining the Q matrix, permutations are taken in the position of the columns in order for the resulting rows in the R matrix to be ordered according to their signal to interference ratio. The K matrix’s quasi-band structure is exploited in order to reduce, as much as possible, the computational complexity in terms of the number of Givens rotations required for the calculation of the QR decomposition.

To accomplish the mentioned decomposition according to the zero-forcing (ZF) criterion, the following extended matrix is defined:

(31) $$\underline{K}=\left[K|v\right],$$

to which a sequence of unit rotations is applied. When the minimum mean square error (MMSE) criterion is used, the extended matrix is constructed including the noise statistics as follows:

(32) $$\underline{K}=\left[\begin{array}{c} K\;v\\ \sigma_{w}^{2}I\;0 \end{array}\right].$$

The advantage of using unit rotations in the orthogonalization process to obtain the QR decomposition is that it conserves the original energy of all the elements of the original K matrix, maintaining a dynamic range of all the variables used in the process. This characteristic makes it easier to implement this method in devices in real-time using fixed-point arithmetic. To simplify the notation in the successive process of Givens rotations, the following notation:

(33) $$X^{\left(0\right)}=\underline{K},$$

is defined. Each Givens rotation, described by matrix $\Theta_{j}$ which is calculated to cancel a non-zero element of the $X^{\left(j-1\right)}$ matrix, obtained in the previous iteration. Therefore, the process of generating the upper triangular matrix R requires a sequence of Givens rotations to be applied to the expanded matrices (31) and (32) as follows:

(34) $$X^{\left(N_{D}\right)}=\Theta_{N_{D}}\cdots \Theta_{1}X^{\left(0\right)}.$$

which ultimately yields

(35) $$X_{N_{D}}=\left[\begin{array}{c} R\;Q^{H}v \end{array}\right]=\left[\begin{array}{c} R\;\tilde{v} \end{array}\right],$$

for the ZF criteria case; for the MMSE criterion the following representation is obtained:

(36) $$X_{N_{D}}=\left[\begin{array}{c} \begin{array}{c} R\;Q_{1}^{H}v \end{array}\\ \begin{array}{c} 0\;Q_{2}^{H}v \end{array} \end{array}\right]=\left[\begin{array}{c} \begin{array}{c} R\;\tilde{v} \end{array}\\ 0\;{\tilde{v}}_{1} \end{array}\right].$$

The detailed description of the Sorted QR algorithm is described in [27], with the only difference of omitting the rotation when a null element is found in the truncated channel matrix K.

### 4.2. Two Approaches for Non-Linear Data Detection

Once the preprocessing of the received signal, and the QR decomposition of the estimated channel matrix, have been completed, the next step is to perform data detection. For accomplishing this task, this work considered the following two non-linear detectors:

#### 4.2.1. OSIC Detection

The ordered successive interference cancellation (OSIC) algorithm is an effective method to carry out the cancellation of the ICI. This method is suitable to apply to the proposed model in Equation (26) combined with the QR decomposition described above, allowing the sub-optimal detection of the data with very low computational complexity. Substituting this decomposition in Equation (26), it results in:

(37) $$v=\mathrm{QR}P^{T}\chi +z_{D};$$

pre-multiplying both sides of this equation by $Q^{H}$ leads to the following system of equations:

(38) $$\tilde{v}=RP^{T}\chi +Q^{H}z_{D},$$

(39) $$\tilde{v}=R\tilde{\chi }+z_{D};$$

where $\tilde{v}=Q^{H}v$, and $\tilde{\chi }=P^{T}\chi$ is the vector of the precoded data, ordered in decreasing order with respect to its contained energy. The $z_{D}$ noise vector maintains its statistics due to the fact that Q is unitary. Due to the triangular structure of R, the $\tilde{v}$ vector elements can be expressed individually as:

(40) $$[\tilde{v}{]}_{j}={\left[R\right]}_{j,j}{\left[\chi \right]}_{j}+\sum_{i=j+1}^{N_{D}}{\left[R\right]}_{j,i}{\left[\chi \right]}_{i}+{\left[z\right]}_{j};$$

where the notations ${\left[\cdot \right]}_{a}$ and ${\left[\cdot \right]}_{a,b}$ indicate the *a*-th element of the vector and the *b*-th element of the *a*-th row of the matrix, respectively. This way, the detection of each of the data signal can be obtained iteratively using the following expression:

(41) $${\left[\hat{\chi }\right]}_{N_{D}}=Q\left\{\frac{{\left[\tilde{v}\right]}_{j}}{{\left[R\right]}_{j,j}}\right\},$$

(42) $${\left[\hat{\chi }\right]}_{j}=Q\left\{\frac{{\left[\tilde{v}\right]}_{j}-\sum_{i=j}^{N_{D}}{\left[R\right]}_{j,i}{\left[\hat{\chi }\right]}_{i}}{{\left[R\right]}_{j,j}}\right\},j=\left\{N_{D}-1,\cdots ,1\right\};$$

where the operator Q{·} is a decision operator that maps its arguments to the closest point in the constellation Ω used by the transmitter. Assuming that at each iteration, the previous decisions are correct, then the interference of the previously detected symbols can be subtracted from the current symbol to be detected.

#### 4.2.2. Near ML Detection

The ML detection using QR decomposition can be reformulated in the following way:

(43) $$\begin{array}{cc} \hat{\chi } & =\underset{\chi \in \Omega^{N_{D}}}{\mathrm{argmin}}{∥\tilde{v}-R\chi ∥}^{2}. \end{array}$$

**Figure 4:**
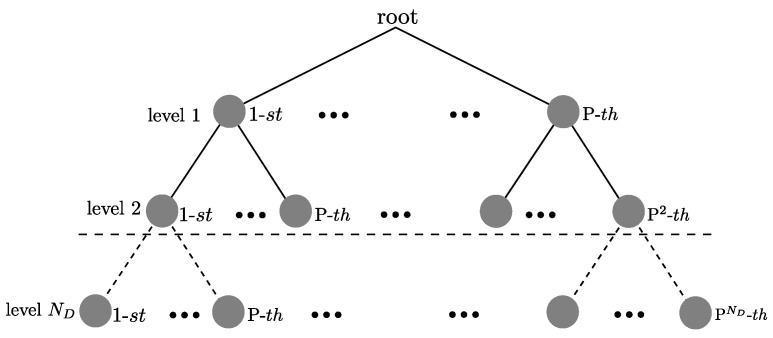
The tree-structured ML solution search.

The search for the ML solution of the $\hat{\chi }$ vector based on the criterion established in Equation (43) can be reflected graphically in the construction of the tree shown in Figure 4. At the *n*-th level there are $q=N_{c}^{N_{D}-n+1}$ possible candidates for ${\left[\hat{\chi }\right]}_{n}$, where $n=\left\{N_{D},N_{D}-1,\cdots ,1\right\}$. The total minimum metric needed to determine the vector with the minimum distance in accordance to Equation (43) is defined as:

(44) $$d\left(\hat{\chi }\right)=\left\{\sum_{j=1}^{N_{D}}∥{\left[\tilde{v}\right]}_{j}-\sum_{i=j}^{N_{D}}{\left[R\right]}_{j,i}{\left[\hat{\chi }\right]}_{i}{∥}^{2}\right\},$$

where:

(45) $$\hat{\chi }=\left\{{\hat{\chi }}_{1},{\hat{\chi }}_{2},\cdots ,{\hat{\chi }}_{N_{D}}\right\},$$

corresponds to the trajectory that forms the nodes at the *n* levels of the tree. The trajectory minimizes the distance calculated in the Equation (44). The symbol vector χ is defined to the *n*-th level as:

(46) $$\chi =\left\{\chi_{N_{D}},\cdots ,\chi_{n}\right\},$$

with $N_{D}-n+1$ length. Adjusting the Equation (44) to calculate the partial metric for the χ vector, the modification is defined as:

(47) $$d_{n}\left(\chi \right)=\left\{\sum_{j=n}^{N_{D}}∥{\left[\tilde{v}\right]}_{j}-\sum_{i=j}^{N_{D}}{\left[R\right]}_{j,i}{\left[\chi \right]}_{i}{∥}^{2}\right\},$$

where $d_{n}\left(\chi \right)$ is the value obtained from the accumulated branch metric of the ${\left[\chi \right]}_{n}$ node that has a ${\left[\chi \right]}_{N_{D}}\ldots ,{\left[\chi \right]}_{n+1}$ as its predecessor nodes. This distance represents the addition of all of the branch metrics from the root ($N_{D}$ node) to the node indicated at the *n*-th level.

**Figure 5:**
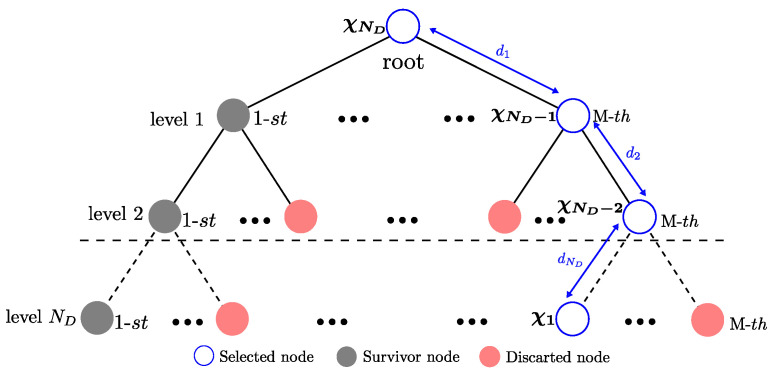
The tree-structured Near ML search proposed.

The QRD-M algorithm based on the QR decomposition reaches a performance in terms of BER, similar to the ML detector [27]. The QRD-M algorithm is a transversal tree search algorithm. At the *n*-th level, the algorithm maintains *M* possible candidates before selecting the symbol. Subsequently, the decision of the $\hat{\chi }$ vector is performed once the $N_{D}$ levels have been processed. The search process in the QRD-M algorithm, used to detect the symbols, is run sequentially and is initiated at the last level ($n=N_{D}$). The algorithm calculates the metric $d_{n}\left(\chi \right)$, defined in Equation (47), for all the possible values of $\chi_{i}\in \Omega$. The distances and the nodes are then arranged in ascending order, and only the *M* nodes with the least distance are maintained while the rest are discarded. The same procedure is used for the next level and continues until the first level (n=1) is processed. The performance of the QRD-M algorithm depends on the established value of $M\leq N_{c}$; a higher value makes it more probable that the optimal branch is included in the branches selected at the *n*-th level. In the search process at each one of the *n*-th levels, the tree extends to $p=MN_{c}$ branches, and their corresponding Euclidean distance is calculated in order to select the surviving *M* branches at the *n*-th level, as illustrated in Figure 5. This value is much lower than the number of branches $q=N_{c}^{N_{D}-k+1}$ that are required in the ML algorithm. Additionally, in our proposal, we introduce heuristics so that the number of surviving *M* branches per level can be adapted. This value is adjusted during the search process run at each level of the tree. With this modification, the detector complexity is variable, and for a high signal to noise ratio the savings in computational complexity of the detector are significant, maintaining the detector’s performance close to that of the ML detector in terms of BER.

Exploiting the R matrix structure, the estimation of the last level ${\left[\chi \right]}_{N_{D}}$ does not depend on the symbols ${\left[\chi \right]}_{j}$, for $1\leq j\leq N_{D}-1$. An exhaustive search is done of ${\left[\chi \right]}_{N_{D}}$, the distances are calculated using Equation (47) for the $N_{c}$ constellation points, such that ${\left[\chi \right]}_{N_{D}}^{\left(\beta \right)}\in \Omega$ for $1\leq \beta \leq N_{c}$. It is defined $d\left(\beta \right)=d\left({\left[\chi \right]}_{N_{D}}^{\left(\beta \right)}\right)=∥{\left[\tilde{v}\right]}_{N_{D}}-{\left[R\right]}_{N_{D},N_{D}}{\left[\chi \right]}_{N_{D}}^{\beta }{∥}^{2}$. The vector elements d(β) are arranged in ascending order and stored in the vector $\mathrm{dist}=\left\{{\left[d\right]}_{1}\leq {\left[d\right]}_{2}\leq \cdots {\left[d\right]}_{N_{c}}\right\}$, where the smallest subindex corresponds to the least vector distance d(β), which corresponds to the ordered set of symbols $\mathrm{pos}=\left\{\chi_{N_{D}}^{\left(1\right)}\;\chi_{N_{D}}^{\left(2\right)}\;\cdots \;\chi_{N_{D}}^{\left(N_{c}\right)}\right\}$ in d(β) terms. The smallest super index in $\chi_{N_{D}}^{\left(\beta \right)}$ indicates the constellation symbol Ω with the least distance in d(β). The symbol search process is continued $\left\{{\hat{\chi }}_{1},\cdots ,{\hat{\chi }}_{N_{D}-1}\right\}$ using the QRD-M algorithm described previously assuming that ${\left[\chi \right]}_{N_{D}}=\chi_{N_{D}}^{\left(1\right)}$. The search result is stored in the vector ${\hat{\chi }}^{\left(1\right)}=\left\{\chi_{1}^{\left(1\right)},\cdots ,\chi_{N_{D}}^{\left(1\right)}\right\}$, with a distance calculated using Equation (44), $d^{\left(1\right)}=\sum_{j=1}^{N_{D}}∥{\left[\tilde{v}\right]}_{j}-\sum_{i=j}^{N_{D}}{\left[R\right]}_{j,i}{\left[{\hat{\chi }}^{\left(1\right)}\right]}_{i}{∥}^{2}$, where the super indexes of $d^{\left(1\right)}$ and ${\hat{\chi }}^{\left(1\right)}$ correspond to the assumption ${\left[\chi \right]}_{N_{D}}=\chi_{N_{D}}^{\left(1\right)}$. A new search is run in the tree and ${\left[\chi \right]}_{N_{D}}=\chi_{N_{D}}^{\left(2\right)}$ is established only if it meets the following condition: ${\left[d\right]}_{2}<d^{\left(1\right)}$ (the first phase demonstrates that $d^{\left(\beta^{*}\right)}=d^{\left(1\right)}$). In case it is not met the significant condition that this search has an initial distance greater than $d^{\left(\beta^{*}\right)}$. The complexity of the optimal vector search for $\hat{\chi }$ in the tree can be reduced further by calculating the partial distance metric to the *n*-th level defined in Equation (47) $d_{n}^{\left(\beta \right)}=\left\{\sum_{j=n}^{N_{D}}∥{\left[\tilde{v}\right]}_{j}-\sum_{i=j}^{N_{D}}{\left[R\right]}_{j,i}{\left[\chi \right]}_{i}{∥}^{2}\right\}$. Additionally, if $d_{n}^{\left(\beta \right)}\geq d^{\left(\beta^{*}\right)}$ is met, the search is cancelled at that level and restarted with the next phase assuming that ${\left[\chi \right]}_{N_{D}}=\chi_{N_{D}}^{\left(\beta +1\right)}$. The algorithm finalizes the $\hat{\chi }$ optimal vector search process when $d^{\left(\beta^{*}\right)}\leq {\left[d\right]}_{\beta }$. A complete description of the Near ML V2V algorithm that includes the previously described procedure is presented in Algorithm 1.
**Algorithm 1:** Recursive near ML detector for V2V system.
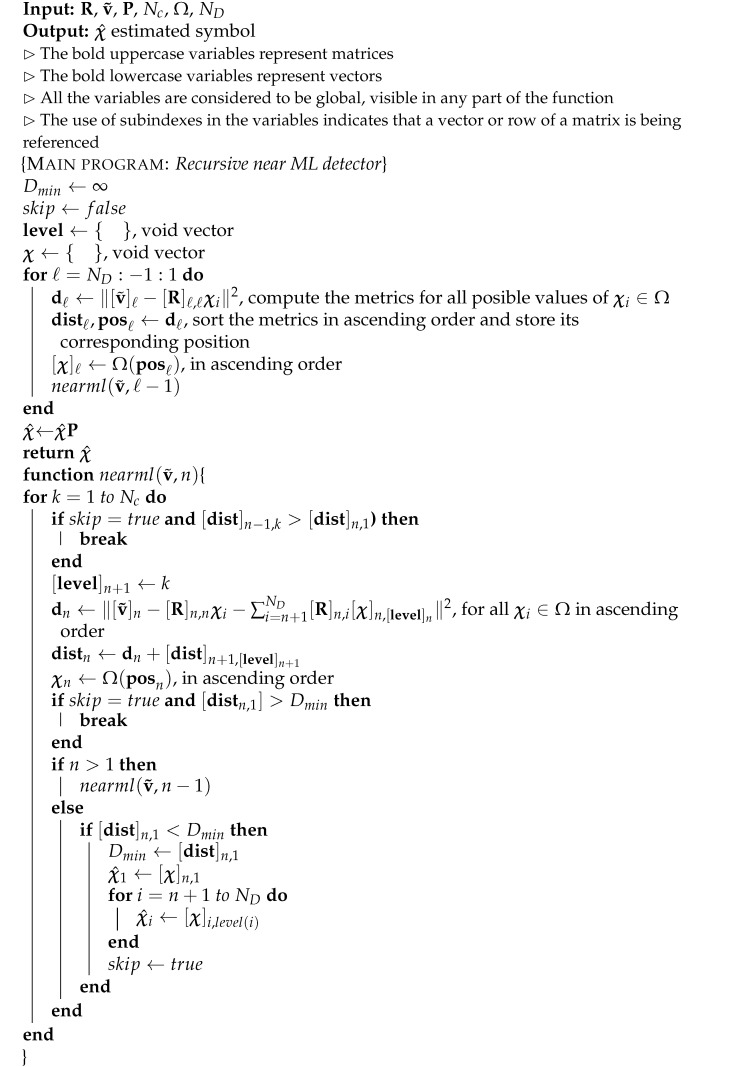


## 5. Computational Structure

**Figure 6:**
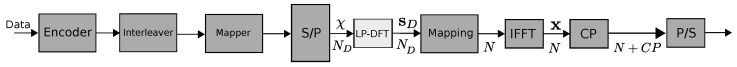
Model of the LP-OFDM transmitter.

**Figure 7:**
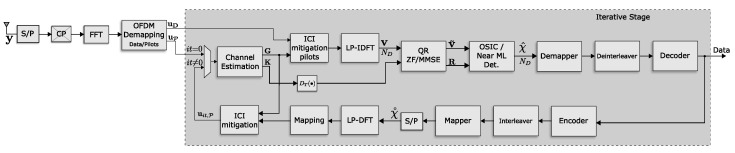
Schematic structure of the 802.11p link-level receiver proposed with no-linear detection and iterative ICI cancellation.

The architecture of the transmitter and the receiver are summarized in Figure 6 and Figure 7, respectively. The transmitter conserves the structure of a conventional OFDM system for the standard 802.11p. The only difference is the DFTS block, applied to the data before the OFDM modulation is completed. The receiver is composed of four main stages. First, the conventional OFDM demodulation is executed with the help of the FFT block, followed by the demapping of the OFDM symbols. The second stage consists of estimating the channel with the use of the pilot symbols. The channel estimator block has the channel matrices G and K as output, necessary to carry out the ICI mitigation and data estimation, respectively. In the third stage, the $D_{T}$ truncation is carried out once the *B* bands of the matrix K are selected. Afterward, the QR decomposition of the truncated matrix K under the ZF or MMSE criterion is carried out. Next, the vector $\tilde{v}$ is calculated by multiplying v and $Q^{H}$. The following step is to carry out either OSIC or Near-ML non-linear detection. At the final stage, bit error correction is performed on the estimated vector $\overset{˚}{\chi }$. The estimated vector enters a feedback loop where ICI mitigation is performed for the data subcarriers. This process reduces the channel estimation error and improves the detection of the data. The process is repeated until the number of iterations configured in the receiver is executed.

## 6. Computational Complexity

The computation complexity is first presented for the QR decomposition; it is given in terms of O and verified by counting the number of complex operations, where Givens rotations are implemented using conventional arithmetic. The parameters considered for the system are:

- An OFDM system with $N_{D}=48$ data subcarriers;
- 4−QAM modulation scheme with unitary average power per symbol;
- $N_{c}=4$ is the maximum number of surviving branches per level in the Near ML V2V algorithm;
- The dimensions of $X_{N_{D}}$ is 48×49 for the ZF criterion and 96×49 for the MMSE criterion;
- The algorithms were performed for 2000 channel realizations, and the complex operations employed were counted independently for each algorithm and finally averaged;
- Based on simulations results, it was determined the number B=27 bands for truncating matrix $\underline{K}$.

**Figure 8:**
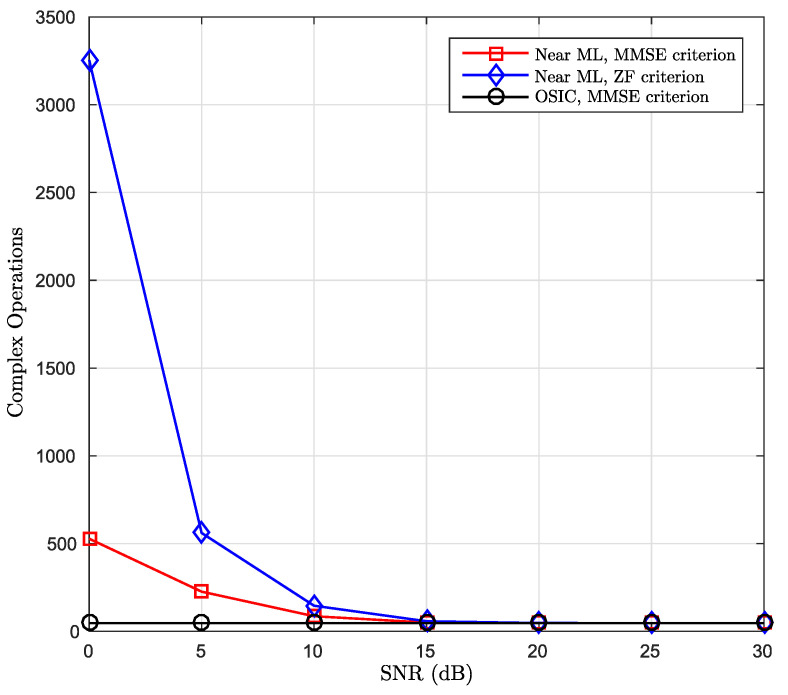
Computational complexity of OSIC and Near ML detectors.

As can be observed in Table 1, the complexity obtained in this proposal, in terms of complex operations, is in quadratic order with respect to the *B* number of bands and in linear order with respect to the $N_{D}$ number of data subcarriers. Systems with similar orders of complexity can be found in [8,14], but the work proposed in this paper provides a significant reduction in the complexity required for the conventional MMSE linear criterion, which is reported in the literature in $O(N_{D}^{3})$ order. A system that uses the real model and LDL decomposition with a complexity similar to $O(B^{2}N_{D})$ was proposed in [8] for an OFDM system in doubly dispersive channels. However, that work does not achieve to improving the performance in terms of BER when compared to the conventional MMSE detection. In the case of the OSIC and Near ML detector complexity, the necessary complex operations were assessed for the detection of $N_{D}$ symbols transmitted by the system. The number of required complex operations was counted depending on the signal-to-noise ratio, both for the ZF criterion and for the MMSE. The results of these simulations are presented in Figure 8.

**Figure 9:**
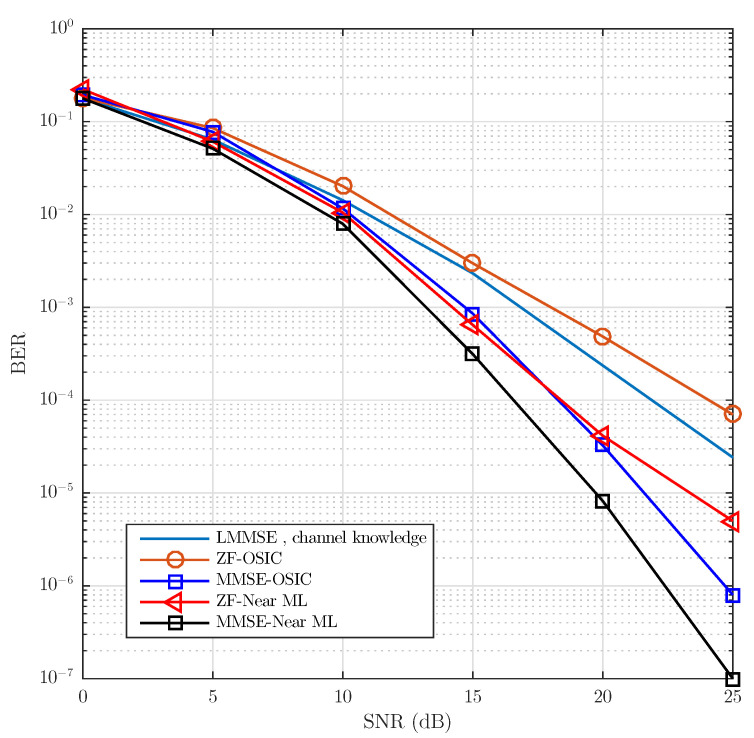
BER vs. SNR comparison without convolutional encoder of the LMMSE with channel knowledge vs. the proposed OSIC and Near ML detections using QR decomposition with ZF and MMSE as criteria. The channel model uses a Jake’s Doppler profile for each channel tap. The power delay profile is exponentially decaying with RMS delay spread of 0.4 μs and frequency Doppler spread of 1 kHz modeling a Rayleigh fading NLOS scenario.

Figure 8 demonstrates the complexity in terms of complex operations required for both proposed detectors. For the case of the OSIC detector the complexity, using the MMSE criterion in the calculation of the QR decomposition, significantly reduces the complexity of the data detection task. For the Near ML detector, none of the two mentioned criteria for QR decomposition is affecting the complexity of finding the optimal vector. The complexity in the OSIC detector is constant and it does not depend on the receiver’s SNR. This approach presents the lowest complexity but its performance is not the best, as shown in Figure 9. In the case of the Near ML detector, Figure 8 shows that its complexity tends towards a low constant value starting at an SNR of 15 dB, where it achieves a very similar value to the complexity obtained by the OSIC detector. On the other hand, its performance, in terms of BER, is quite close to the ML detector, as will be discussed in detail in the following section.

Table 2 summarizes the complexity required by the most representative detection algorithms used in doubly selective channels (DSC). The main objective is to provide the reader with an overview of the computational complexity of the compared approaches. Table 2 shows the closeness in computational cost of Near ML detection compared to Sorted OSIC detection. The LMMSE detection requires approximately three times the computational cost of the OSIC and Near ML detection. The proposed algorithms achieve a cost reduction in $10^{25}$ order compared with the Full-ML detection.

## 7. Simulations Results

The numerical results presented below were obtained from a simulator implemented in Matlab-Simulink, replicating compatible simulation environments with the 802.11p link [11]. The blocks that describe the internal signal processing algorithms considered for the transmitter and the iterative receiver were discussed in Section 5.

The standard 802.11p uses a bandwidth of BW=10 Mhz, a cyclic prefix containing CP = 16 samples, due to which the system can absorb a maximum tolerable delay of 1.6 μs in the duration of the CIR from the V2V channel. Of the 64 subcarriers that compose an OFDM symbol, 48 are used for the data transmitting. This allows the use of eight modulation schemes which permit the handling of transmission speeds between 3 and 27 Mbps and a frame length of $L_{F}=37$ OFDM signals.

In this article, we present results using the following parameters: a 4-QAM modulation scheme, $N_{c}=4$ for the Near ML search tree, convolutional coding of length $L_{c}=7$, a code rate of Rc=1/2 and B=27 main diagonals, with $\lambda =8,\lambda_{c}=5$. The channel was implemented using the filtered method explained in [28]. The channel power delay profile (PDP) consists of six uncorrelated paths defined by $p\left(\tau \right)=\delta \left(\tau -m\tau_{0}\right)e^{\frac{\tau }{10\tau_{0}}}$ where 0<τ<1.6μs, $\tau_{0}=0.1$ μs and m={0,1,…,}. The parameters that were used to generate the V2V channel are reported in [3], where V2V channel models consider vehicle velocities of v=100 Km/h. The number of OFDM symbols transmitted by each evaluated SNR level is equal to $1.6\times 10^{5}$.

**Figure 10:**
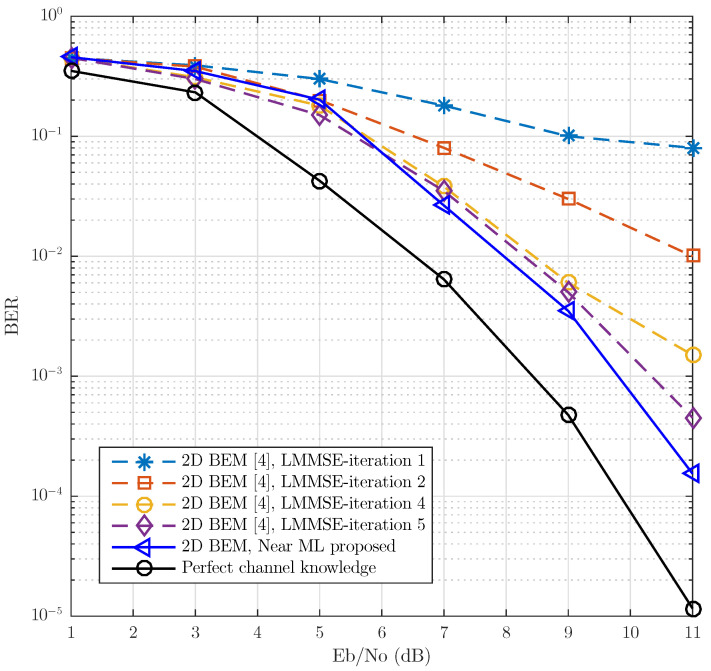
BER vs. Eb/No comparison of the Near ML detection proposed and LMMSE detection for a frame length $L_{F}=37$ and full 802.11p compliant (no postamble). The channel model uses a Jake’s Doppler profile for each channel tap. The power delay profile is exponentially decaying with RMS delay spread of 0.4 μs and frequency Doppler spread of 1 kHz modeling a Rayleigh fading NLOS scenario.

Figure 10 shows the BER vs. $E_{b}/N_{0}$, under a vehicular scenario with NLOS (no-line-of-sight). To emulate this scenario, a channel with Rayleigh fading was considered with a power delay profile, decreasing exponentially with root mean square (RMS) delay time $\tau_{RMS}=0.4$μs and $f_{D}=1$ kHz. This model is similar to the one called “RTV-Expressway” described in [2]. The dotted lines show the system performance with linear detectors, using the pilot assignment shown in Figure 1 for the iterative estimation of the channel. The solid line is assigned to the performance of our proposed system with Near ML non-linear detection. The tests show that the linear detection approach needs at least three iterations in order for a floor error not to be found. In the case of the proposed Near ML non-linear detection, no iteration is needed to achieve this. It can be seen that the proposed Near ML detection largely surpasses the LMMSE detection approach proposed in [3].

In order to more clearly quantify the Near ML and OSIC detection performance with respect to the DFTS-OFDM-LMMSE detection, tests were completed without the use of the convolutional encoder. Additionally, both the OSIC and Near ML detection were evaluated using QR decomposition with ZF and MMSE as criteria. Figure 9 shows the BER vs. SNR comparison of the proposed algorithms that includes frequency dispersion. With the exception of the ZF-OSIC detection, the proposed algorithms present better performance compared with LMMSE detection with ideal channel. In one particular case above SNR = 15 dB, the MMSE-OSIC suboptimal detection and the MMSE-Near ML detection surpass the LMMSE detection by 2.5 and 5 dB, respectively. It is important to mention that the tendency shown by both proposed detectors does not exhibit the undesirable error floor.

**Figure 11:**
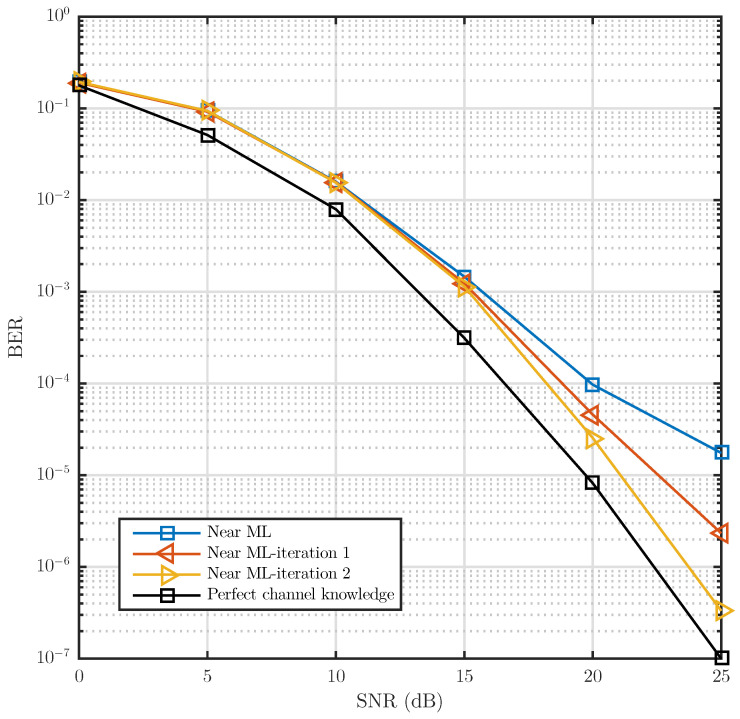
BER vs. SNR for the iteration {1,2} of the Near-ML detection proposed without convolutional encoder, using QR decomposition with MMSE as criteria. The channel model uses a Jake’s Doppler profile for each channel tap. The power delay profile is exponentially decaying with RMS delay spread of 0.4 μs and frequency Doppler spread of 1 kHz modeling a Rayleigh fading NLOS scenario.

Figure 11 shows the performance of the proposed iterative receiver. With just two iterations completed in the proposed receiver, a performance similar to the ideal channel, in terms of BER, is achieved. This represents a substantial decrease in the number of iterations required by the receivers reported in state of the art, which require at least five iterations to achieve a performance similar to the ICI-free condition.

## 8. Conclusions

This article has presented a low computational complexity receiver that achieves both the extraction of frequency diversity, as well as efficient ICI mitigation in V2V communication systems. It was shown that reception in challenging high-mobility environments can be achieved with the proposed scheme, also exhibiting a manageable computational complexity. The proposal consists of an efficient treatment of the data frequency dispersion, a time-varying channel estimation using the two dimensional basic expansion model, and sub-optimal non-linear detectors. The results show a performance close to the one obtained by the ML algorithm. Furthermore, with only two iterations, it was shown that with true low complexity, the proposed system performed equally or better, in terms of BER, than other approximations presented as the state of the art, confirming its capacity to exploit the available frequency diversity in doubly selective channels.

## Figures and Tables

**Figure 2:**
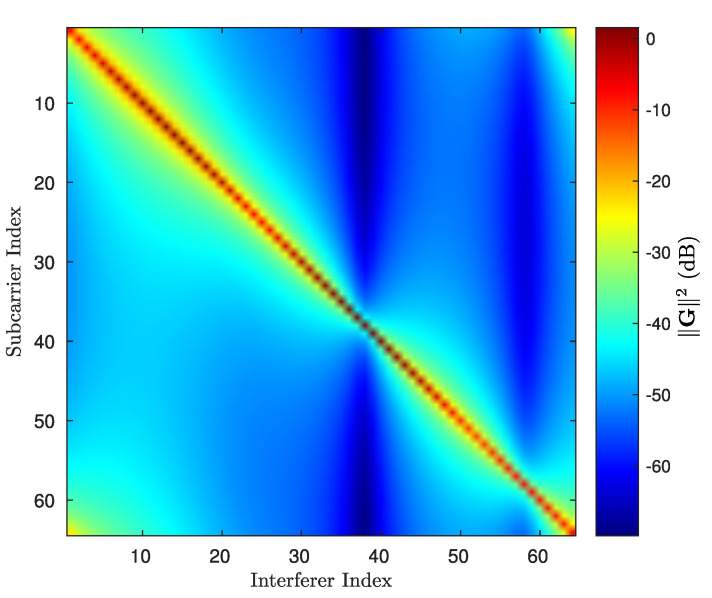
Example of the quasi-band structure of channel frequency matrix ${∥G∥}^{2}$ in dB, for a V2V scenario with RMS delay spread of 0.4 μs and frequency Doppler spread of 1 kHz, modeling a Rayleigh fading NLOS scenario.

**Table 1:** Computational complexity required for sorted-QR decomposition.

QR Decomposition	Number of Rotations with Banded Matrix	Number of Rotations with Full Matrix	Complexity
ZF	37,900	114,020	$O(B^{2}N_{D})$
MMSE	59,650	172,780	$O(2B^{2}N_{D})$

**Table 2:** Computational complexity in terms of complex products per OFDM symbol required for signal detection, considering one iteration with $N_{c}=4,B=27,\Omega =4$, and SNR =15 dB.

Method	Complexity	Normalized Cost	
		$N_{D}=48$	$N_{D}=512$
ML	$O(\Omega^{N_{D}}N_{D})$	5.43 $\times 10^{25}$	Inf
LMMSE	$O(N_{D}^{3})$	1.5802	179.7970
Near ML Proposed	$O(\left(2B^{2}+N_{c}^{2}\right)N_{D})$	1.0110	1.0110
OSIC Proposed	$O(2B^{2}N_{D})$	1	1

## Data Availability

The data presented in this study are available on request from the corresponding author.
